# Development of dodecahedron type MCP detector for SEM toward full energy range and solid angle electron detection

**DOI:** 10.1093/jmicro/dfag008

**Published:** 2026-02-17

**Authors:** Yuto Yanagihara, Yuanzhao Yao, Kazuhiro Kumagai, Takashi Sekiguchi

**Affiliations:** The Graduate School for the Creation of New Photonic Industries, 1955-1 Kurematsuchō, Chūō-ku, Hamamatsu, Shizuoka 431-1202, Japan; Institute of Pure and Applied Sciences, University of Tsukuba, 1-1-1 Tennodai, Tsukuba, Ibaraki 305-8573, Japan; Institute of Pure and Applied Sciences, University of Tsukuba, 1-1-1 Tennodai, Tsukuba, Ibaraki 305-8573, Japan; Hokuriku Digital Manufacturing Center, National Institute of Advanced Industrial Science and Technology (AIST), 10-2 Harue-cho Edome-Kamiyamato, Sakai, Fukui 919-0462, Japan; Institute of Pure and Applied Sciences, University of Tsukuba, 1-1-1 Tennodai, Tsukuba, Ibaraki 305-8573, Japan

**Keywords:** SEM, MCP, dodecahedron, energy- and angle-selective detection

## Abstract

A detector was developed for scanning electron microscopy (SEM) in which microchannel plates (MCPs) were mounted on the facets of a regular dodecahedron. This detector enables the detection of electrons emitted in multiple directions. By placing a bias grid in front of each MCP, both backscattered electrons (BSE) and secondary electrons (SE) can be discriminated and detected. Using this detector, images were obtained by detecting electrons emitted in the direction normal to the sample surface (Top0), in two different oblique upward directions (Up1 and Up2), and in an oblique downward direction (Down6), to evaluate the emission-angle dependence including components emitted toward the lower hemisphere. Three specimens were examined: (i) a Cu plate with fine curtain-like surface waviness, (ii) a Cu–Al eutectic microstructure and (iii) a three-dimensional stainless-steel sphere. For the Cu plate, surface corrugations were emphasized in the Up1/Up2 BSE images, whereas Top0 showed nearly uniform contrast. For the Cu–Al specimen, Top0 primarily provided compositional contrast, while the Up1/Up2 highlighted interfacial regions due to illumination-effect-like directional acceptance. For the stainless-steel sphere, obliquely downward BSE were clearly detected with Down6, indicating the usefulness of the downward channel for three-dimensional geometries. Since this regular dodecahedral detector operates without electric or magnetic fields that could distort electron trajectories, it enables analysis of the energy and emission-angle dependence of emitted electrons. This design uses identical MCP detectors at multiple viewing directions, which simplifies signal handling and facilitates multi-view analysis of direction- and energy-resolved signals.

## Teaser

A regular-dodecahedral detector for SEM was developed using microchannel plates. Bias-grid filtering discriminates secondary and backscattered electrons. Several imaging examples demonstrate the versatility of the detector.

## Introduction

In a conventional out-lens scanning electron microscope (SEM), backscattered electrons (BSEs) are detected by a Si photodiode (Si-PD) detector placed just above the specimen, whereas secondary electrons (SEs) are detected by an Everhart-Thornley (ET) detector mounted on the chamber wall [1–3]. High-energy BSE travel nearly straight, while low-energy SE are drawn toward the ET detector by its electric field [1–4]. Because the detection efficiency of a Si-PD is low for electrons below 1 keV, the Si-PD in practice collects mainly BSE [5]. Thus, even though the emission direction is not discriminated, energy separation generally yields SE and BSE images.

By contrast, recent SEM schemes equipped with in-lens (through-the-lens) detectors pull emitted electrons into the objective-lens system and select them using electron optics and bias grids [6–9]; since lens structures and discrimination methods vary across instruments, it is often not self-evident whether a given image is BSE- or SE-origin [7]. Therefore, this article reports an electron detector that discriminates SE and BSE from the specimen by energy and by emission angle. We experimentally verify energy- and angle-selective discrimination in an out-lens SEM by extending the principle demonstrated in [5] to a regular-dodecahedral multi-face arrangement. The focus of this article is the experimental verification and the image-interpretation benefits using representative specimens; detailed fabrication of each MCP module is described in [5]. We developed an MCP-based detector for out-lens SEM that can discriminate and collect electrons by energy and by emission direction. This enables the separation and interpretation of contributions such as surface relief (topography), composition contrast and surface potential in BSE/SE images.

The detection system mounts compact MCPs on each face of a regular dodecahedron that partitions the full solid angle into twelve sectors, with the specimen placed at the center. This placement controls the distance and geometric conditions to each face and enables a solid-angle-based comparison. A grid, acting as a high-pass filter for emitted electrons, was installed in front of each MCP to perform energy discrimination [5,10]. In this manner, contributions such as surface relief, composition and surface potential embedded in BSE/SE images can be separated and interpreted.

In this article, we present images obtained at an accelerating voltage of 15 kV from multiple detector faces under controlled bias-grid high-pass filtering conditions. We discuss how energy- and angle-selective discrimination can aid image interpretation using three representative specimens (a Cu surface with wave curtain-like waviness, a Cu–Al eutectic alloy and a stainless-steel sphere). The discussion is limited to SE and BSE. The present paper focuses on experimental verification and application to SEM imaging.

## Experimental

**Fig. 1.**
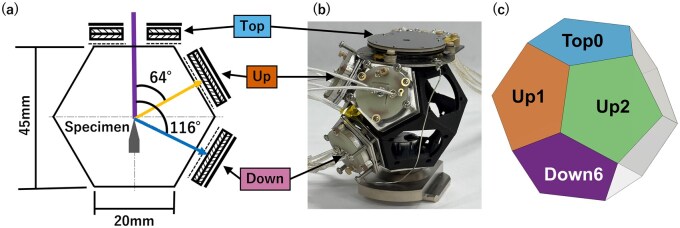
Configuration of the dodecahedral MCP detector: (a) schematic geometry, (b) photograph, and (c) face naming for the detector channels used in this study. MCPs, microchannel plates.

Figure 1 shows a schematic and a photograph of the detection system. Up to twelve microchannel plates (MCPs) can be mounted on the faces of a regular dodecahedral frame with an edge length of 20 mm. The faces are designated Top0, Up1–Up5, Down6–10 and Bottom11. MCPs manufactured by Hamamatsu Photonics K.K. were used. Top0 has an effective diameter of Φ20 mm and a central beam hole of Φ6 mm (dead area Φ8 mm). The Up, Down and Bottom positions have an effective diameter of Φ15 mm. In front of each MCP’s signal-input area, a three-grid bias assembly was installed to function as a high-pass filter [5,10]. The MCP plane is located 26 mm from the dodecahedron center. The solid angle subtended by the detectors is 0.35 sr for Top0 and 0.24 sr for the others; summing over all 12 faces yields 2.99 sr, i.e. about 24% of the full solid angle. These solid angles were estimated geometrically from the effective MCP entrance areas at a specimen–MCP distance of 26 mm (Top0: effective φ20 mm excluding the central dead area of φ8 mm; others: effective φ15 mm) and then summed over the 12 faces. The total is 2.99 sr, corresponding to about 24% of 4π steradians.

In the present experiments we used Top0, Up1, Up2 and Down6. MCPs were installed only on these faces. Taking the electron-beam incidence direction as the zenith, the central pointing directions (Θ,Φ) of each detector are listed in Table 1. The specimen was placed at the center of the dodecahedron; for the spherical sample, a needle holder was used to avoid casting shadows. The MCP has peak sensitivity around 500 eV and detects electrons from a few eV up to several tens of keV [5,11]. The energy-dependent response of the MCP and the detection principle are described in [5]. In the present paper, we focus on comparative image interpretation rather than absolute quantification of electron yield. Therefore, we do not claim absolute intensity ratios (e.g. SE-to-BSE or between detectors) without dedicated sensitivity calibration.

**Table 1.** Geometrical parameters of the dodecahedral detector; Central directions (Θ, Φ) and geometric solid angles for the detector faces used in this study

Detector	Θ [deg]	Φ [deg]	Solid Angle [sr]
Top0	0°	0°	0.35
Up1	64°	0°	0.25
Up2	64°	72°	0.25
Down6	116°	36°	0.25

The detection system was mounted on a Schottky field-emission SEM (FE-SEM; SU7000; Hitachi High-Tech, Tokyo, Japan). Imaging conditions were 15 kV accelerating voltage and 1 nA beam current. The SU7000 booster setting was ON. The working distance (WD) was 37 mm for the Cu and Cu–Al specimens. For the stainless-steel sphere, WD was 27 mm due to the specimen height on a needle holder. Systematic evaluation of WD dependence is beyond the scope of this article and will be addressed in future work. High-pass-filtered images were obtained by applying 0 V, −50 V and −1 kV to the bias grid. The two outer grids were kept at ground potential and acted as electrostatic shields. A negative voltage was applied to the middle grid to form a retarding potential; electrons with energies lower than the applied voltage were rejected at the grid, while electrons with energies higher than the applied voltage passed through the grid and reached the MCP.

In this work, images were acquired at bias-grid voltages of 0 V, −50 V and −1 kV. The 0 V image contains both SE and BSE components. The −50 V condition rejects electrons below 50 eV; we use the difference image (0 V minus −50 V) as the SE image. The −1 kV condition rejects electrons below 1 keV and is used as the BSE image under that cut condition.

JMONSEL is a Monte Carlo electron-trajectory simulation code based on the NIST Monte Carlo model [12]. In this study, JMONSEL was used to qualitatively interpret the detector-direction-dependent BSE contrast observed near the Cu–Al interface.

**Fig. 2.**
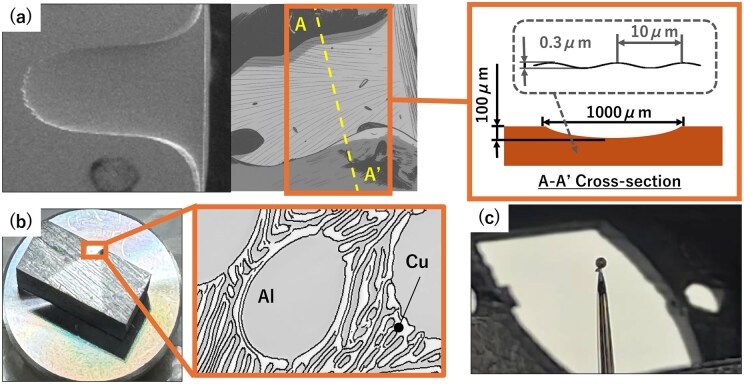
Specimens and imaging configuration: (a) Ar-ion-milled Cu surface, (b) Cu–Al eutectic microstructure, and (c) stainless-steel sphere mounted on a needle holder.

The specimens used are shown in Fig. 2; purposes and details are as follows:

1. Relief contrast (Fig. 2a): a Cu surface with “curtain” waviness made by cross-section ion milling, exhibiting a period of 10 µm, amplitude of 0.3 µm and a maximum local tilt of ±11°.
2. Composition contrast (Fig. 2b): a Cu–Al eutectic microstructure prepared by bonding Cu and Al plates followed by heat treatment.
3. Three-dimensional detection (Fig. 2c): a stainless-steel sphere of 0.5 mm diameter mounted on a needle holder to eliminate shadowing and detect direction-dependent electrons emitted from a three-dimensional specimen, including electrons emitted in an oblique downward direction (Down6), i.e. toward the lower hemisphere.

## Results and discussion

### Relief contrast on Cu

**Fig. 3.**
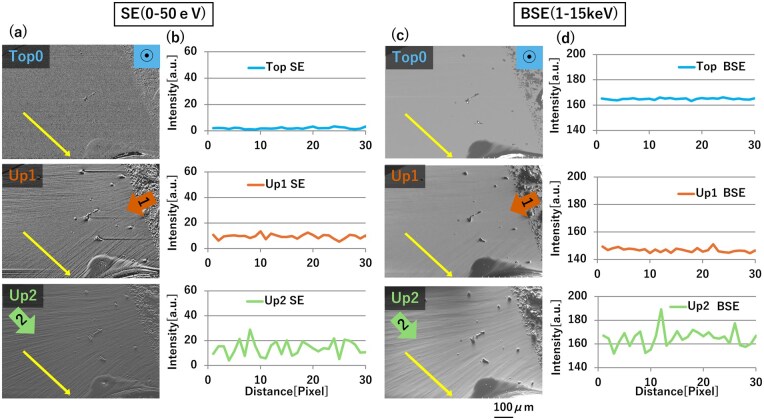
SE (0–50 eV) and BSE (1–15 keV) images of an Ar-ion-milled Cu surface acquired with Top0, Up1, and Up2. (b, d) Line profiles along the arrows in (a, c). BSEs, backscattered electrons; SEs, secondary electrons.

Figure 3 shows Ar-ion-milled Cu observed with the Top0, Up1 and Up2 detectors. The ion beam was incident from the 2 o’clock direction on the right (i.e. toward Up1), so the ridges and troughs of the surface corrugation extend along that direction. The left column shows SE images and the right BSE images. With Top0, both SE and BSE images exhibit nearly uniform brightness across the milled surface, with no clear features apart from overall intensity differences. With Up1, faint streaks corresponding to the surface waviness are visible in the SE image, whereas the BSE image shows at most weak modulation of the surface waviness under this viewing geometry and mainly reveals dark specks from small surface debris. With Up2, streaks corresponding to the surface waviness are clearly visible in both SE and BSE images, and the black–white contrast of debris is enhanced. In the Up1 SE image, an elongated streak appears to the right of each speck – namely along the beam-scan direction. This is not observed in the BSE image, nor with Top0 or Up2, and is likely a scan-direction-dependent streak artifact due to charging that appears when the scan direction is close to the detector viewing direction. This attribution is based on the fact that it is observed only in the Up1 SE image under this geometry and is absent in the corresponding BSE image and in the Top0/Up2 views. Along the direction of the arrow in Fig. 3, the fluctuations of the SE signal increase as the direction of the milling-formed fringes gradually deviates from the direction of Up1. This indicates a pronounced illumination-effect dependence in the SE signal under the present geometry.

In summary, SE and BSE emitted near the surface normal are insensitive to the waviness. For oblique emission, the relief is reproduced most strongly when the detector view is perpendicular to the striation; when it is close to parallel (as in the Up1 configuration here), the modulation becomes weak – particularly in BSE – and may be hardly visible. Thus, while SE is more sensitive to relief overall, BSE better reflects the directionality of the relief. This is also evident from the line profiles attached to the images. A systematic evaluation of WD dependence – especially for low-energy SE detection – is left for future work. Under the present configuration, the Top0 SE component was weak; therefore, the SE/BSE balance is discussed only qualitatively. Because no dedicated sensitivity calibration was performed, the apparent SE/BSE balance in the images is not a calibrated electron-count ratio. Accordingly, these results demonstrate that the interpretation of SE/BSE images can change markedly with detector viewing direction, and that multi-directional acquisition provides practical cues for separating relief- and composition-related contrast in an out-lens SEM.

### Density contrast in Cu–Al

**Fig. 4.**
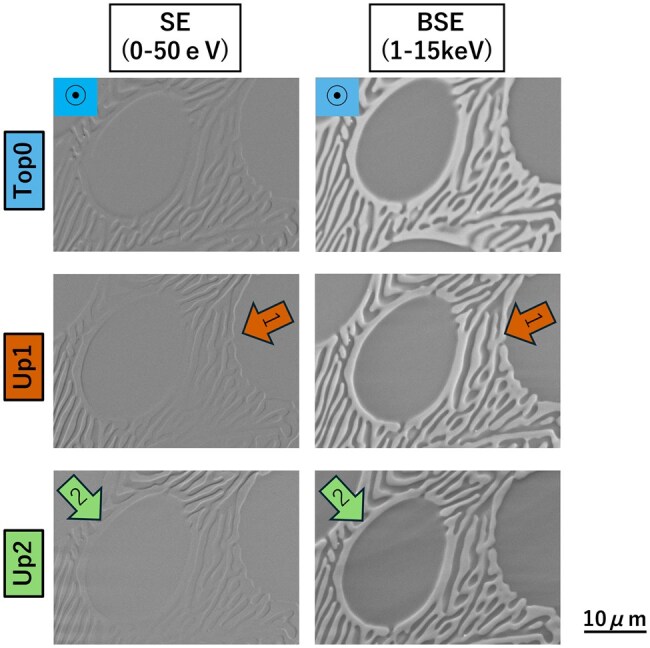
SE/BSE images of the Cu–Al eutectic microstructure acquired with Top0, Up1, and Up2 detectors. BSEs, backscattered electrons; SEs, secondary electrons.

Figure 4 shows a Cu–Al eutectic microstructure recorded with Top0, Up1 and Up2. In the BSE images, Cu appears bright and Al dark for all three detectors. Because the effective diameter of the MCP differs between Top and Up, a strict intensity comparison is difficult, but the overall level is comparable among them. In Up1 and Up2, edge contrast at Cu boundaries is emphasized. By contrast, the SE images are darker than the BSE images and show only a slight brightening over Cu regions. We use JMONSEL to qualitatively interpret detector-direction-dependent BSE contrast near the Cu–Al interface.

**Fig. 5.**
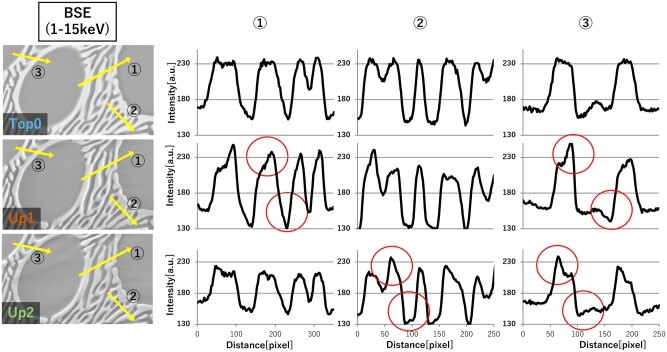
BSE line profiles across selected Cu–Al interfaces (①–③) for Top0, Up1, and Up2. BSEs, backscattered electrons.

**Fig. 6.**
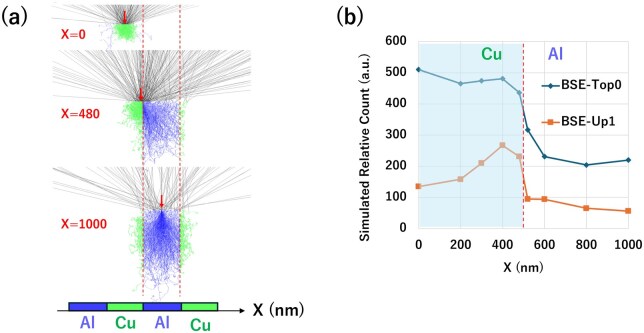
(a) JMONSEL simulation results of electron trajectories in the Cu region and Al region for beams incident at different positions near the Cu–Al interface. Black trajectories represent electrons emitted into the vacuum; red arrows indicate the incident positions. (b) Simulated relative BSE counts collected by the Top0 and Up1 detector acceptances as a function of the incident coordinate *x* (15 keV, normal incidence). BSEs, backscattered electrons.

Compared with Top0, Cu–Al edges are enhanced in Up1/Up2. To visualize this, three line segments (①–③) are drawn in Fig. 5, and the BSE intensity variations along them are plotted in Fig. 5. With Top0, both the Cu “peaks” and Al “minima” are left–right symmetric for all three lines, with no edge enhancement. In Up1, lines ① and ③ show enhancement on the right side: the Cu peak grows and the Al minimum deepens; line ② remains nearly symmetric. In Up2, lines ② and ③ show enhancement on the left side: the Cu peak increases and the Al minimum decreases. As discussed by Reimer [3], after primary-electron incidence, elastic and inelastic scattering produce an asymmetric spread in Cu and Al, leading to detector-direction-dependent variations in BSE yield (schematized in Fig. 6). The Top detector collects electrons nearly symmetrically from the Cu and Al sides across the boundary, so edges are not emphasized. For the Up detectors, when an edge is oriented nearly perpendicular to the detector view, the detector-side edge is emphasized – Cu edges become brighter while Al edges become darker – whereas edges parallel to the detector view are not emphasized. While BSE images primarily reflect composition, mixed two-phase systems may exhibit unexpected contrast patterns, which must be considered in interpretation.

To explain our experimental observations, a Monte Carlo simulation of electron scattering near the Cu–Al interface was performed using JMONSEL. The model consists of a flat Cu–Al eutectic microstructure under normal incidence of a 15 keV electron beam. The widths of the Cu and Al regions are each 1 µm, consistent with our experimental sample. As shown in Fig.  6a, the coordinate origin (*x* = 0) is defined at the center of the Cu region. When the beam is incident near the interface (*x* = 480 nm), the scattering trajectories become asymmetric in Cu and Al. Moreover, the density of emitted electron trajectories toward the right side is higher than toward the left. The simulated relative BSE counts collected by the Top0 and Up1 detector acceptances are plotted as a function of the incident coordinate in Fig.  6a. The Up1 signal exhibits a peak near the interface, whereas the Top0 signal shows a step-like profile without a peak feature. This behavior is consistent with our experimental results for Top0 and Up1 along the direction ① in Fig.  5.

### Three-dimensional detection of a stainless-steel sphere

**Fig. 7.**
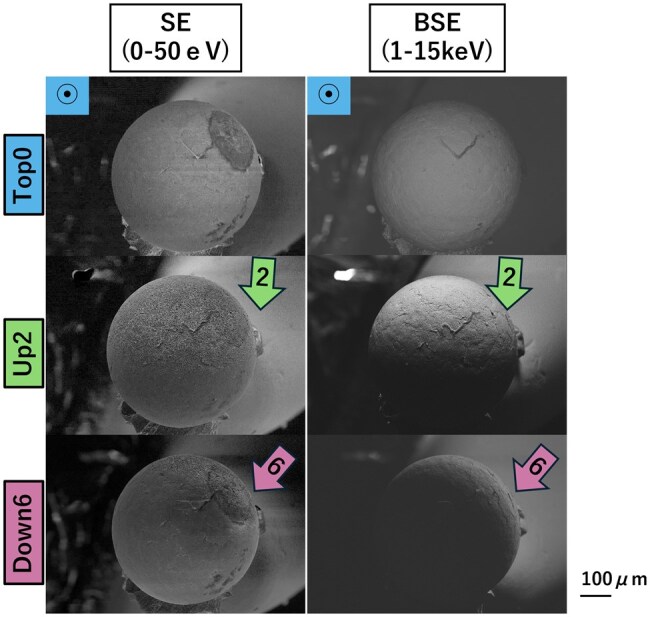
SE/BSE images of a stainless-steel sphere acquired with Top0, Up2, Down6 detectors (SE: 0–50 eV; BSE: 1–15 keV). BSEs, backscattered electrons; SEs, secondary electrons.

In this article, “three-dimensional detection” refers to detecting emitted electrons from a three-dimensional specimen from multiple directions, including an oblique downward direction (Down6). Figure 7 shows SE and BSE images of a stainless-steel sphere acquired with Top0, Up2 and Down6. For Top0, the SE image is uniformly bright over the sphere, whereas the BSE image is bright at the center and becomes darker toward the periphery. The uniformity of the SE image arises from cancelation between the edge effect (∝ 1/cos *θ*) and the emission-angle dependence (∝ cos *θ*), where *θ* is the angle between primary electron beam and surface normal. The BSE distribution reflects the emission-angle dependence (∝ cos *θ*). These trends agree with our previous work [13]. With Up2, both SE and BSE images show a shift of the brightness maximum toward the detector direction – an illumination-effect-like behavior – more pronounced for BSE than for SE. With Down6, this feature becomes even stronger. The SE image shows a faint, nearly uniform background intensity, which may include contributions from stray electrons (often referred to as SE3). In this article, we use “SE3” to denote such a weak, spatially broad background component in the SE images. The main focus of this study is the separation and interpretation of specimen-origin SE (SE1/SE2) and BSE; SE3 is mentioned only in the context of this observed background. The bright rim on the detector-side circumference in the BSE image reflects the BSE intensity maximum appearing around ∼2*θ*. Following Reimer [2,3], the dominant emission direction of BSE is approximately twice the local incidence angle (specular-like behavior); therefore, for a detector with a fixed viewing direction Θ_det (given by the detector central direction in Table 1), the condition Θ_det ≈ 2*θ* is satisfied near the detector-side rim of the sphere, producing the bright rim.

In summary, the dodecahedral detector enables multi-directional SE and BSE imaging of a three-dimensional specimen, including an oblique downward direction (Down6). The results in Fig. 7 show that the SE/BSE intensity distribution on the sphere shifts toward the detector direction and becomes more pronounced for BSE, demonstrating direction-dependent detection in a practical SEM configuration.

Down/Bottom detectors are not optimal for flat, bulk specimens, but they can be advantageous for thin films and non-flat topographic surfaces (e.g. stepped or patterned structures) because they preferentially collect obliquely emitted electrons. This geometry is therefore expected to enhance sensitivity to surface relief and edge-related contrast. In this study, we focus on demonstrating multi-directional detection; quantitative multi-view analysis and reconstruction will be addressed in future work.

### Current status, issues and prospects of the dodecahedral MCP arrangement

In the present measurement, the SE and BSE signal levels depend on the detector configuration and bias-grid settings; therefore, we do not discuss an absolute SE-to-BSE intensity ratio because no dedicated sensitivity calibration was performed. Low-energy SE detection is sensitive to residual electric fields and charging. In addition, the mesh/grid can scatter a fraction of signal electrons.

At present, the maximum detectable solid angle fraction is ∼24%. This value is obtained by summing the geometric solid angles of the effective MCP entrance areas at the specimen–MCP distance of 26 mm. Specifically, the effective entrance area is defined from the MCP diameters used in this work (Top0: 20 mm excluding the 8 mm central dead area; other faces: 15 mm). Using these effective areas, the per-face solid angles are calculated geometrically at r = 26 mm and summed over the 12 faces to give 2.99 sr; dividing by 4π sr yields 24%.

The 50% value is a design projection under the same dodecahedral geometry and distance, not an experimentally measured value. It assumes that the inactive/dead area around each MCP entrance is reduced so that the effective entrance area on each face increases while keeping the same face orientations and specimen–MCP distance. Under this assumption, the summed solid angle increases in proportion to the total effective entrance area, giving 50%. Increasing the detectable solid-angle fraction beyond this level is difficult without changing the frame geometry because the face area and the required mechanical and electrical margins limit the maximum realizable entrance area on each face. Realizing an “electron integrating sphere” remains a challenge for future work. An “electron integrating sphere” refers to a detector concept that collects emitted electrons from as many directions as possible, approaching 4π steradians, to minimize directional bias.

## Concluding remarks

We experimentally demonstrated practical discrimination and interpretation of SE and BSE in a conventional out-lens SEM using a regular-dodecahedral MCP-based electron detector with a wide solid-angle acceptance (2.99 sr, ∼24% of 4π) over a broad energy range. Electrons emitted from the specimen at the dodecahedron center are direction-sorted and detected by MCPs on the twelve faces, with a three-grid bias assembly acting as a high-pass filter for energy discrimination. SE and BSE images were separated using the bias-grid filtering and image-differencing procedure. Using this detector, we conducted (i) relief imaging of a Cu plate, (ii) compositional imaging of a Cu–Al eutectic microstructure and (iii) three-dimensional detection of a stainless-steel sphere. These results show the image-interpretation benefits of energy- and angle-selective SE/BSE discrimination. Analysis of the images acquired with this detector clarifies the energy and emission-direction dependences of SE and BSE, advancing the physical interpretation of SE and BSE images. Ref. [5] demonstrated energy-selective SE/BSE imaging using a thin MCP detector with a bias-grid filter and image subtraction. The present paper extends this principle to a multi-face dodecahedral arrangement and shows, through three representative specimens, that direction-dependent signals acquired from multiple faces provide practical cues for interpreting relief- and composition-related contrast in SE/BSE images.

## Data Availability

The data that support the findings of this study are available from the corresponding author upon reasonable request.
